# A new source of data to inform policy on chronic diseases

**DOI:** 10.3389/fpubh.2026.1904713

**Published:** 2026-07-22

**Authors:** Ray Campbell, George Ochoa

**Affiliations:** FAIR Health, New York, NY, United States

**Keywords:** chronic diseases, commercially insured, data, FAIR Health Atlas, policy

## Abstract

Chronic diseases are the nation’s leading cause of disability and death. To guide policy discussions about chronic diseases, data are essential but have been lacking on the commercially insured. FAIR Health is introducing Atlas, a free data source that addresses this need. Based on the nation’s largest collection of recent commercial claims, FAIR Health Atlas visualizes chronic disease data across all fifty states and Washington, DC, revealing areas facing health challenges. Results using FAIR Health Atlas concern the commercially insured. For example, Atlas data show that West Virginia was the state with the highest prevalence of diabetes in 2025—14,406.45 per 100,000. Colorado had the lowest prevalence that year, at 6,547.79 per 100,000. Breast cancer prevalence in West Virginia in 2025, however, was lower than in Colorado. By enabling insights into chronic diseases in the commercially insured population, FAIR Health Atlas can contribute to policy discussions concerning such diseases.

## 1 Introduction

Chronic diseases are the nation’s leading cause of disability and death (1). As such, they are central to public policy issues. States, policy makers and analysts, in their varied efforts to address health disparities, have focused on chronic disease. Discussions range widely about how to mitigate the effects of social determinants of health on chronic illness, and how to interpret geographic disparities. Rising costs, access to affordable care, and care coordination are issues frequently linked to chronic diseases.

To guide policy discussions on these and other issues, data are essential. Most data on chronic diseases have come from Medicare and Medicaid (2), but the majority of working-age adults—including those with chronic diseases—have commercial coverage. Indeed, many chronic diseases first occur in adults aged 18 to 64 (3), the period when they are most likely to be commercially insured. Data on chronic diseases in the commercially insured can thus help inform policy decisions affecting this population.

FAIR Health is pleased to introduce Atlas (4), a new, freely available data source that addresses this need. FAIR Health Atlas visualizes chronic disease data across all fifty states and Washington, DC, revealing the areas facing the greatest health challenges.

**Figure 1:**
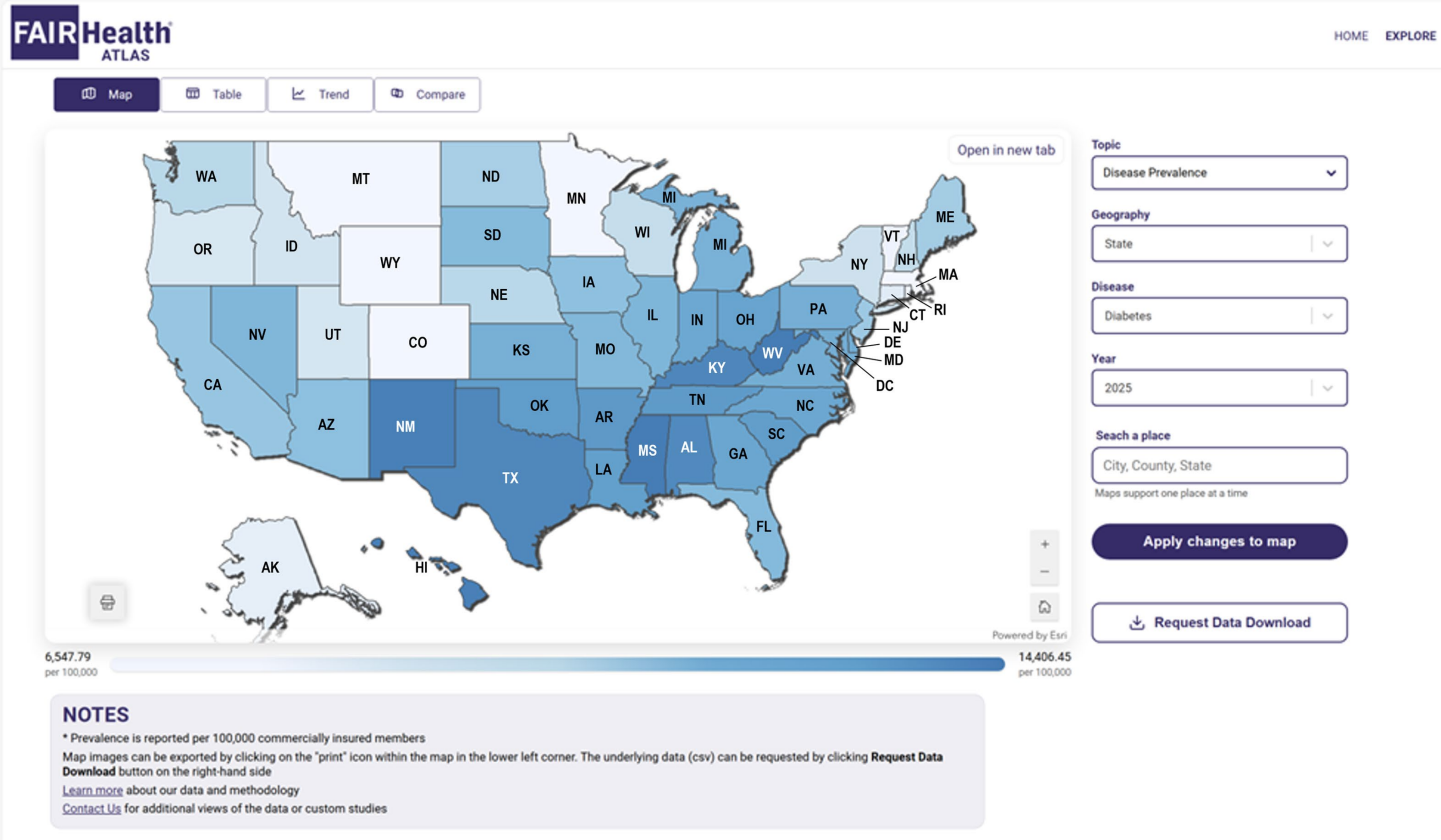
Diabetes prevalence by state, 2025. Source: FAIR Health.

**Figure 2:**
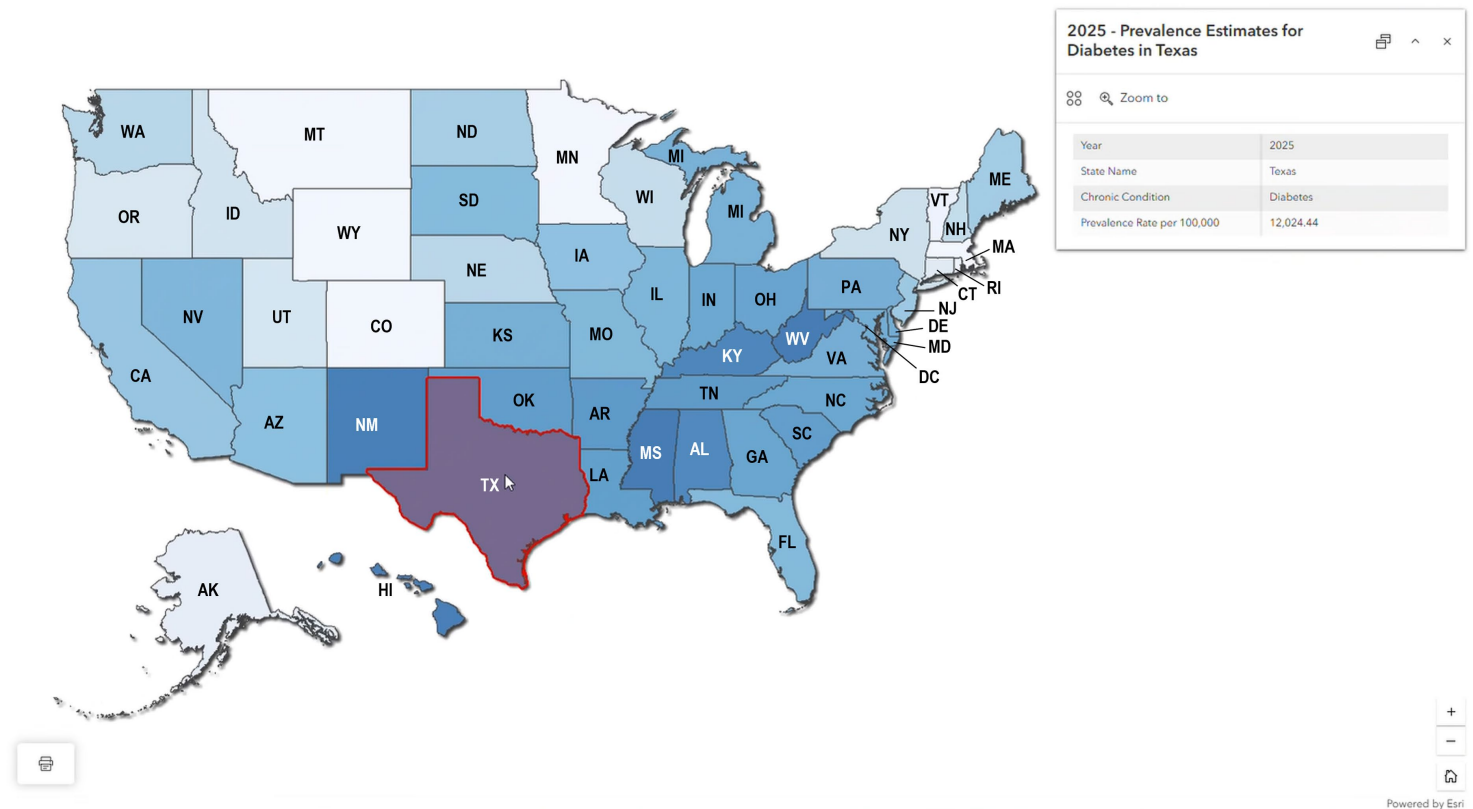
Diabetes prevalence in Texas, 2025. Source: FAIR Health.

Visitors to FAIR Health Atlas can explore disease prevalence. Data can be viewed as a map (see Figure 1), table, or trend chart. Users can search locations, switch between state and county views, change disease and year selections, and download data for further analysis. Clicking on a state or county triggers a pop-up window that shows that location’s name and the relevant condition’s prevalence there (Figure 2).

## 2 The methods behind FAIR Health Atlas

The disease prevalence data in FAIR Health Atlas come from the nation’s largest repository of commercial healthcare claims, growing at a rate of more than four billion claim records a year. FAIR Health, a national, independent nonprofit, maintains that database as part of its mission to supply objective, unbiased information for all stakeholders to improve healthcare quality, access, and affordability. Atlas will give policy makers, public health professionals, nonprofits, and researchers access to information about chronic condition prevalence and health disparities in the United States.

We estimate chronic disease prevalence by first counting the number of commercially insured individuals in our database who have a claim containing a chronic condition diagnosis code, as defined by the Centers for Medicare and Medicaid Services’ Chronic Conditions Data Warehouse (2). We use census data to weight those results, adjusting the counts included in our data to reflect the entire commercially insured population. (If an individual does not seek any medical care within the year, or does not have insurance from one of our data contributors, they will not appear in our database.)

Once we have counts for each service zip code, we use a nearest neighbors technique to pool data from the surrounding areas to make the estimates more robust. We draw a circular disk around each service zip code and expand that disk in one-mile increments until it contains at least 100,000 members. Once this threshold is passed, we calculate the prevalence by dividing the adjusted count of commercially insured individuals with that chronic disease in the disk by the total commercially insured population in the disk, using estimates reported by the US Census Bureau’s Small Area Health Insurance Estimates program (5). This creates a smoothed surface of chronic disease estimates that is more accurate and robust to variations in service zip code population size. From these smoothed disk estimates, we then calculate the county- and state-level estimates through population-weighted averages. We present these data scaled to rates per 100,000 commercially insured members to correspond with standard epidemiologic reporting and for ease in the comparison across regions of varying populations.

## 3 How FAIR Health Atlas advances research

Our 2026 white paper *Chronic Conditions in the United States: A Study of Commercial Claims* (6) incorporates analyses that can be done with FAIR Health Atlas, as well as other analyses (such as those related to cost data). In addition, here are a few results that were obtained just by using FAIR Health Atlas, all concerning the commercially insured population.

West Virginia was the state with the highest prevalence of diabetes in 2025—14,406.45 per 100,000. Colorado had the lowest prevalence that year, at 6,547.79 per 100,000. The association of West Virginia with higher disease prevalence than Colorado, however, was not consistent across all chronic diseases. The prevalence of breast cancer in West Virginia in 2025, for example, was 1,225.71 per 100,000. In Colorado, the prevalence of breast cancer that year was higher—1,440.53 per 100,000.

FAIR Health Atlas can spotlight counties as well as states. For example, an examination of New York County (Manhattan) shows that the prevalence of hypertension in that county in 2025 was 12,169.36 per 100,000. But the prevalence just across the river in Kings County (Brooklyn) was higher, at 18,418.80 per 100,000.

FAIR Health Atlas can also generate trend charts, showing change over time. West Virginia was the state with the highest prevalence of Alzheimer’s disease in 2025. Alzheimer’s disease prevalence, however, declined in West Virginia from 2020 to 2025, from 323.12 per 100,000 in 2020 to 320.18 in 2025.

## 4 Discussion

By enabling insights into chronic diseases in the commercially insured population, FAIR Health Atlas can contribute to the policy discussions concerning such diseases. It is especially useful for aiding conversations about health disparities, whether on a state-by-state or county-by-county basis. Future versions of Atlas may introduce even more capabilities, but what is available in this first iteration is a strong foundation for research that can inform policy.

## Data Availability

Publicly available datasets were analyzed in this study. This data can be found at: https://atlas.fairhealth.org/ [forthcoming September 16, 2026]. Further inquiries can be directed to the corresponding author.
